# Insulin Receptor Expression and Activity in the Brains of Nondiabetic Sporadic Alzheimer's Disease Cases

**DOI:** 10.1155/2012/321280

**Published:** 2012-05-16

**Authors:** Lap Ho, Shrishailam Yemul, Lindsay Knable, Pavel Katsel, Rudy Zhao, Vahram Haroutunian, Giulio Maria Pasinetti

**Affiliations:** ^1^Department of Neurology, Mount Sinai School of Medicine, 1468 Madison Avenue, New York, NY 10029, USA; ^2^Department of Psychiatry, Mount Sinai School of Medicine, 1468 Madison Avenue, New York, NY 10029, USA; ^3^Mental Illness Research, Education and Clinical Center, James J. Peters Veteran Affairs Medical Center, 130 West Kingsbridge Road, Bronx, NY 10468, USA; ^4^Geriatric Research, Education and Clinical Center, James J. Peters Veteran Affairs Medical Center, 130 West Kingsbridge Road, Bronx, NY 10468, USA

## Abstract

We investigated the contents of the insulin receptor-beta subunit (IR**β**) and [Tyr1162/1163]-phosphorylated IR**β** as surrogate indices of total IR content and IR activation in postmortem hippocampal formation brain specimens from nondiabetic sporadic Alzheimer's disease (AD) cases. We found no significant changes in the brain contents of total IR**β** or [Tyr1162/1163]-phosphorylated IR**β**, suggesting normal IR content and activation in the brains of nondiabetic sporadic AD cases. Moreover, total IR**β** and [Tyr1162/1163]-phosphorylated IR**β** levels in the hippocampal formation are not correlated with the severity of amyloid or tau-neuropathology. Exploring the regulation of glycogen synthase kinase 3 (GSK3) *α*/**β**, key IR-signaling components, we observed significantly lower levels of total GSK3 *α*/**β** in brain specimens from nondiabetic AD cases, suggesting that impaired IR signaling mechanisms might contribute to the onset and/or progression of AD dementia. Outcomes from our study support the development of insulin-sensitizing therapeutic strategies to stimulate downstream IR signaling in nondiabetic AD cases.

## 1. Introduction

Evidence from numerous epidemiological studies indicates that type 2 diabetes (T2D, a noninsulin-dependent form of diabetes mellitus) is associated with a two- to three-fold increase in the relative risk for Alzheimer's disease (AD), independent of the risk for vascular dementia [1–9]. Experimental evidence suggests that abnormalities in insulin metabolism under diabetic conditions could mechanistically influence the onset of AD via modulation of the synthesis and degradation of amyloidogenic beta-amyloid (A*β*) peptides. For example, insulin itself may significantly promote A*β* accumulation by accelerating amyloid precursor protein/A*β* trafficking from the *trans*-Golgi network, a major cellular site for A*β* generation, to the plasma membrane [10]. Moreover, elevated circulating insulin contents under diabetic conditions may also promote amyloid accumulation by direct competition with A*β* for the insulin-degrading enzyme (IDE), and therefore may limit A*β* degradation by IDE [11, 12].

In addition to the direct roles of insulin and IDE, accumulating evidence shows that under diabetic conditions, impairments in certain insulin receptor- (IR-) responsive cellular signaling pathways might also mechanistically promote AD-related neuropathology and cognitive deterioration [13–18]. Building on this observation, a recent hypothesis implicates impaired insulin signaling in the brain as a common underlying cause of sporadic AD, regardless of diabetic or nondiabetic status [19].

Cellular insulin signaling is initiated by the coupling of extracellular insulin with the insulin receptor in the plasma membrane, which leads to IR activation and subsequent promotion of cellular IR-signaling processes [20]. Despite the central role of IR activation in cellular IR-signaling processes, there is limited and conflicting information available on the regulation and activity of IR in the brains of sporadic AD cases. In particular, Frölich et al. [21] reported significantly increased IR-binding activity in the brains of sporadic AD cases. In contrast, Steen et al. [19] and Rivera et al. [22] observed that AD is associated with significantly reduced IR contents and “IR activity” (i.e., IR tyrosine phosphorylation) in the brain. Moloney et al. [23] recently reported no change in the levels of total IR*α* and IR*β* subunits, but found an aberrant subcellular distribution of IR*α* and IR*β* in temporal cortex specimens from cases characterized by severe AD neuropathology, suggesting the presence of compromised IR signaling in surviving AD neurons. None of the studies indicate the diabetic status of the study subjects. A recent study by Liu et al. [18] reported no change in the total IR*β* subunit level in postmortem frontal cortex specimens from AD cases without diabetes, but there is little information given on the criteria by which the absence of diabetes was determined, and there is no information regarding the activation status of the insulin receptor.

Accumulating epidemiological and experimental evidence suggests that in the AD brain, impairments in select cellular signaling pathways associated with (but not necessarily limited to) IR signaling might mechanistically promote AD phenotypes [2, 3, 6, 7, 14–17]. Among these, impaired glycogen synthase kinase 3 (GSK3) function in the AD brain has been considered pivotal for disease development [24–27]. GSK3 is a ubiquitously expressed, highly conserved serine/threonine kinase involved in numerous cellular processes [28]. There are two mammalian GSK3 isoforms, GSK3*α* and GSK3*β*, with GSK3*β* being particularly abundant in the central nervous system. GSK3*α* and *β* are constitutively active, but are inactivated by IR-responsive Akt-mediated phosphorylation at [Ser21]-GSK3*α* and [Ser9]-GSK*β*, respectively [28]. Some studies argue that overactivity of GSK3 plays a critical role in the pathogenesis of both sporadic and familial AD (for review, see [29]). Accordingly, GSK3 hyperactivity may contribute to AD by increasing tau hyperphosphorylation, promoting A*β* production, and/or stimulating brain inflammatory responses [29]. However, contrary to this hypothesis there are studies that show evidence of reduced total GSK3 contents and activity in the AD brain [24, 25]. In particular, a study by Baum et al. [24] revealed significantly reduced contents of total (nonphosphorylated (active) and phosphorylated (inactive)) GSK3*α* and GSK3*β* in the AD brain. A second study by Griffin et al. [25] observed significantly reduced contents of GSK3*β*, coupled with a significantly elevated ratio of ser9-phospho-GSK3*β*/total GSK3*β*, implicating inactivation of GSK3*β* in AD compared to control brain specimens. None of the studies on the regulation of GSK3 in the AD brain indicate the diabetic status of the study subjects. In a more recent paper, Liu et al. [18] reported no significant change in total GSK3*β* or phosphorylated GSK3*β* protein levels in the brains of nondiabetic, sporadic AD cases.

While T2D is a risk factor for AD, there is little information available on the regulation and activity of IR in the AD brain, either in the absence or in the presence of comorbid diabetic conditions. IR is a tetrameric transmembrane receptor comprised of two *α* and two *β* subunits [30]. Insulin binding to IR leads to rapid autophosphorylation of specific tyrosine residues in the IR*β* subunit, which converts IR*β* into a catalytic active conformation that is necessary for IR signal transduction [30]. For example, IR*β* autophosphorylation at Tyr1162/1163 is critical for stabilizing IR*β* in a catalytically active conformation [31]. The present study was designed to explore the regulation of IR contents and IR activation in the brains of nondiabetic AD cases. We assessed the contents of total (nonphosphorylated and phosphorylated) IR*β* and [Tyr1162/1163]-phosphorylated IR*β* as surrogate indices of, respectively, total IR content and IR activation in the brains of nondiabetic AD cases as a function of clinical AD dementia and AD neuropathology. Results from our studies demonstrated that there is no detectable change in IR content and activation in the brain. Nonetheless, we found significantly lower levels of total GSK3*β* protein in the brains of nondiabetic AD cases, suggesting that impaired IR signaling mechanisms might contribute to the onset and/or progression of AD dementia in the absence of diabetes.

## 2. Materials and Methods

### 2.1. Evaluating the Impact of Postmortem Interval on the Detection of Phosphorylated IR*β* in Mouse Brain Specimens

Mice were sacrificed by cervical dislocation and freshly isolated brain specimens were either immediately frozen or stored postmortem for up to 6 hours at room temperature before homogenization for analysis of total and phosphorylated IR*β* contents. Tissue specimens were homogenized in Tris/Triton solution: 250 mM sucrose, 50 mM Tris-HCl (pH 7.4), 1 mM EDTA, 2 mM EGTA, 1% Triton X100 containing 1 mM PMSF and cocktails of proteinase/phosphatase inhibitors (Pierce Biotech Inc, Rockford, IL, USA). Total protein concentration in the tissue homogenates was determined with a CBQCA Quantitation Kit (Molecular Probes Inc, Eugene, OR, USA). Aliquot samples of total protein contents (15 *μ*g) were loaded in triplicates onto pre-cast 8% Precise protein gels (Pierce Biotech Inc, Rockford, IL, USA) under reducing conditions. Electrophoresis and transblotting were performed under standard conditions. Total (nonphosphorylated and phosphorylated) IR*β* and phosphorylated IR*β* were detected, respectively, using mouse monoclonal L55B10 antibodies for total IR*β* and rabbit monoclonal 19H7 antibodies for [Tyr 1150/1151]-phosphorylated IR*β*; both antibody preparations were obtained from Cell Signaling Technology Inc. (Danvers, MA). Image detection was conducted using infrared fluorescence detection (IRDye 680 or 800 goat antiappropriate species IgG, Li-Cor Biosciences, Lincoln, NE, USA) and scanned using the Odyssey Infrared Imaging System (Li-Cor Biosciences, Lincoln, NE, USA). Images were analyzed and quantified using Odyssey software ver.3 (Li-Cor Biosciences, Lincoln, NE, USA).

### 2.2. Patient Selection Criteria

Human postmortem temporal muscle and hippocampal formation specimens from AD and age-matched non-AD cases were obtained from the Alzheimer's Disease Brain Bank of the Mount Sinai School of Medicine [32]. The cases selected had no significant neuropathological features or had only neuropathological features associated with AD [32, 33]. Cognitive status of the cases was assessed based on the cognitive dementia rating (CDR), which is generated using a multistep evaluation of cognitive and functional status during the last 6 months of life, as previously reported [34]. Moreover, only nondiabetic cases were selected for this study; cases with a premorbid history of diabetes were excluded. Diabetic (T2D) or nondiabetic cases were identified using criteria previously described [35, 36]. Our analysis included only cases with no record of diabetes (absence of reported history and failure to meet blood chemistry-based criteria); cases with a premorbid history of diabetes were excluded (i.e., plasma glucose concentration >200 mg/dL, fasting glucose >126 mg/dL, 2-hour plasma glucose > 200 mg/dL during oral glucose test, and impaired fasting glucose was defined as 110–125 mg/dL (6.1–7.0 mmol/L)).

Tissue samples were divided into groups based on their CDR categories as follows. CDR 0: cognitive normal (*n* = 10); CDR 0.5, at high risk of developing AD dementia (*n* = 9); CDR 1, mild AD dementia (*n* = 11); CDR 2, moderate AD dementia (*n* = 13); CDR 5, severe AD dementia (*n* = 19).

### 2.3. Beta-Amyloid and Neurofibrillary Tangle Neuropathology Assessments in Human Brain Specimens

The extent of neuritic plaque (NP) and neurofibrillary tangles (NFTs) staining in the brain (entorhinal cortex) was assessed in accord with the consortium to establish a registry for Alzheimer's disease (CERAD) neuropathologic battery [37]. The density of NPs and NFTs were rated on a 4-point scale: 0, absent; 1, sparse; 3, moderate, and 5, severe. NPs were visualized after either Bielschowsky silver or thioflavin-S staining [38]. Multiple (~5) high power (×200, 0.5-mm) fields were examined in each histological slide from multiple regions according to the CERAD regional sampling scheme. All investigators were masked to the clinical diagnosis of each case until all histological and biochemical analyses were completed and values were assigned to each specimen.

The contents of A*β*_1–40_ and A*β*_1–42_ in the hippocampal formation were assessed as previously described [39]. Briefly, frozen tissue samples were homogenized in a buffer containing 70% formic acid and 100 mmol/L betaine, and soluble A*β*_1–40_ and A*β*_1–42_ were quantified by enzyme-linked immunosorbent assays (ELISAs) using, respectively, synthetic A*β*_1–40_ and A*β*_1–42_ (US Peptides, Fullerton, CA, USA) as standards. Microtiter plates were coated with 2 mg/mL monoclonal antibody 4G8 (Senetek, Maryland Heights, MO, USA), which recognizes an epitope between residues 17 and 20 of A*β*. Unoccupied binding sites on the plates were blocked by incubation with casein. Samples and standards were applied in quadruplicate and incubated for 48 hours at 4°C. After the A*β*_1–40_ and A*β*_1–42_ capture phase, the plates were probed with, respectively, an A*β*_1–40_ or an A*β*_1–42_ C-terminal-specific antibody, followed by incubation with a reporter antibody (alkaline phosphatase-conjugated anti-rabbit IgG, *γ*-chain-specific) (JBL Scientific, San Luis Obispo, CA, USA). The assay was developed using an alkaline phosphatase substrate (Attophos; JBL Scientific), yielding a fluorescent product, and analyzed with a 96-well fluorescence reader (CytoFluor; Millipore, Bedford, MA, USA). All samples were analyzed in the linear range of the ELISA.

### 2.4. Regulation of Total IR*β* Expression and [Tyr1162/1163]-IR*β* Phosphorylation in Human Brain or Temporal Muscle Specimens

Frozen banked tissue (hippocampal formation or temporal muscle) specimens were powderized under liquid nitrogen and were then homogenized in ice-cold cell lysis buffer (20 m Tris/HCl, pH7.5, 150 mM NaCl, 1 mM ECTA, 1 mM EGTA, 1% Triton X-100, 2.5 mM sodium pyrophosphate, 1 mM beta-glycerophosphate, 1 mM Na3VO4, 1 ug/mL leupeptin and 1 mM phenyl sulphonyl fluoride) using a hand held BioVortexer or Pellet Pestle Motor (Kontes, Northbrook, IL, USA) as previously described [14, 39]. The homogenates were sonicated three times for 10 seconds each (Sonic Dismembrator Model 500, Fisher Scientific) and were then centrifuged at 13,000 xg for 15 min. Supernatants were collected and protein concentrations were determined using Bradford protein assays (Bio-Rad laboratories, Hercules, CA, USA). Supernatants (lysates) were stored at −80°C until further analysis.

Total IR*β* protein content was quantified by Western blot analysis. Protein extracts (25 *μ*g) were separated on 10% SDS-PAGE under reducing conditions and transferred to PVDF membranes using 10 mM CAPS pH11, 10% methanol at 4°C. The membranes were blocked with 5% blocking grade nonfat dry milk in 10 mM Tris/HCl pH7.6, 140 mM NaCl, 0.1% Tween-20, before being incubated with a primary anti-IR*β* antibody (rabbit polyclonal IgG, C-19, 1 : 500 dilution; Santa Cruz Biotechnology Inc., Santa Cruz, CA, USA). Membranes were washed and incubated with an HRP-conjugated secondary antibody, washed, and bands were detected using chemiluminescence methodology (Amersham ECL plus western blotting detection system, GE Healthcare, UK) followed by exposure to Kodak X-ray films. Films were scanned and appropriate protein band densities were quantified with Bio-Rad Quantity-One software (Bio-Rad laboratories, Hercules, CA, USA). Assessment of *β*-actin content using a rabbit polyclonal anti-*β*-actin antibody (Sigma, St. Louis, MO, USA) on the same blots served as a loading control.

Assessments of [Tyr1162/1163]-phosphorylated IR*β* protein contents were conducted using a commercial sandwich [Tyr1162/1163]-phosphorylated IR*β* ELISA assay (BioSource International, Inc., Camarillo, CA, USA) that is specific for IR*β* and does not cross-react with IGF-1R*β*. In this study, the [Tyr1162/1163]-phosphorylated IR*β* ELISA was conducted according to the manufacturer's recommendations. A lyophilized lysate from insulin-stimulated human IR transfected Chinese hamster ovary cells provided by the manufacturer served as a quantitative standard; 1 unit of standard is equivalent to the amount of IR [Tyr1162/1163] derived from 0.6 *η*g of IR (*β*-subunit) in transfected Chinese hamster ovary cells stimulated with 100 nM insulin.

### 2.5. Regulation of GSK3 *α*/*β* Expression in Human Brain Specimens

Contents of total GSK3 *α*/*β* [including both phosphorylated (inactive) and nonphosphorylated (active) forms] in hippocampal formation specimens were assessed by western blot. In this study, 25 *μ*g of lysate proteins was assayed using a commercial anti-GSK3 *α*/*β* antibody (mouse monoclonal 1H8 antibody, dilution 1 : 3,500; Calbiochem, San Diego, CA, USA) that simultaneously detects total GSK3*α* and total GSK3*β* (inactive phosphorylated and active nonphosphorylated GSK3 *α*/*β*); identification of GSK3*α* and GSK3*β* is based on their unique molecular sizes: 51 kDa for GSK3*α* and 47 kDa for GSK3*β*.

### 2.6. Statistics

Statistical analysis was performed using the Prism software package (GraphPad Software, Inc, San Diego, CA, USA). Analysis of variance (ANOVA) was used to evaluate differences in mean values among three or more groups, and the Dunnett *t*-test was used to test the significance of the differences in means. One-tailed *t*-tests were used as indicated. Correlation analysis between two variables was done using the Pearson parametric method followed by 2-way analysis of the *P* value.

## 3. Results

### 3.1. Patient Populations

Patient information including age, postmortem interval, gender, and neuropathological findings for cases assessed in this study is summarized in Table 1. Only nondiabetic cases were selected for this study; cases with a premorbid history of diabetes were excluded. Analysis of variance indicated that there were no significant differences among the CDR groups with respect to age (*P* = .40) and postmortem interval (*P* = .82) at the time of death.

### 3.2. Evaluating the Potential Impact of Postmortem Interval on the Detection of Phosphorylated IR*β*

**Figure 1:**
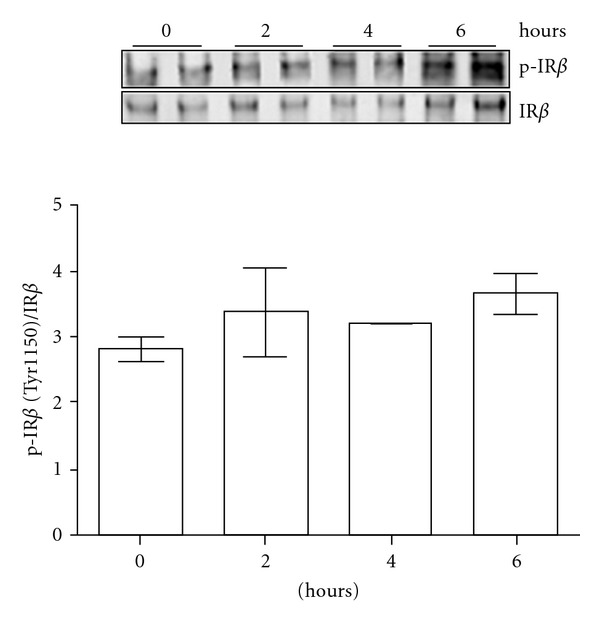
Stability of phosphorylated IR*β* in mouse brain specimens as a function of postmortem interval. Mice were sacrificed and brain specimens were isolated immediately. Freshly isolated mouse brain specimens were either rapidly frozen or were kept at room temperature for up to a 6 hr postmortem interval. Samples were assessed at 2 hr postmortem intervals as indicated. Bar graphs represent the ratio of [Tyr1150/1151]-phosphorylated IR*β*/total IR*β* as mean ± SEM values. ANOVA; *P* = .531; Inset: representative western blot analysis of [Tyr1150/1151]-phosphorylated IR*β* and total IR*β* at different postmortem time intervals as indicated.

Postmortem interval (PMI) is known to affect the phosphorylation status of a number of signaling proteins. For example, Li et al. [40] examined a number of signaling proteins, such as ERK, JNK, RSK, CREB, and ATF-2 proteins, in mouse brain specimens at 0, 8, 24, and 48 hrs postmortem, and demonstrated dramatically reduced contents of phosphorylated species for each of these proteins by 8 hrs postmortem. The cohort of 62 nondiabetic cases selected for our present study were characterized by a relatively shorter than average postmortem interval, ranging from a minimal average postmortem interval of 4.3 ± 0.4 h for the CDR 0.5 cases to a maximal average postmortem interval of 5.44 ± 0.91 h for the CDR 0 cases (Table 1). In a series of control studies using mouse brain specimens, we explored the potential impact of similarly short postmortem intervals on the detection of tyrosine phosphorylated IR*β* in brain specimens. We dissected mouse brain tissue and assessed [Tyr1150/1151]-phosphorylated IR*β* contents from tissue specimens kept at room temperature and found no significant changes in the detection of tyrosine phosphorylated IR*β* levels (normalized to total IR*β*) from mouse brain specimens that were kept at room temperature for up to 6 hrs postmortem (Figure 1). This suggests that the relatively short postmortem intervals that are associated with the human brain specimens used in our present study likely have no appreciable impact on the detection of tyrosine-phosphorylated IR*β* contents from these specimens.

### 3.3. Assessment of Total IR*β* and [Tyr1162/1163]-Phosphorylated IR*β* Contents in the Periphery and in the Brain

We assessed temporal muscle and hippocampal formation specimens from the same cases to explore the regulation of IR in the periphery and in the brain among nondiabetic cases across CDRs. In these studies, Total IR content was assessed by Western blot analysis of total IR*β* peptide contents using a specific antibody that does not cross-react with IGF-1R*β*. The content of [Tyr1162/1163]-phosphorylated IR*β*, assessed using a specific ELISA that does not cross-react with IGF-1R*β*, was used as a surrogate index of IR activation.

**Figure 2:**
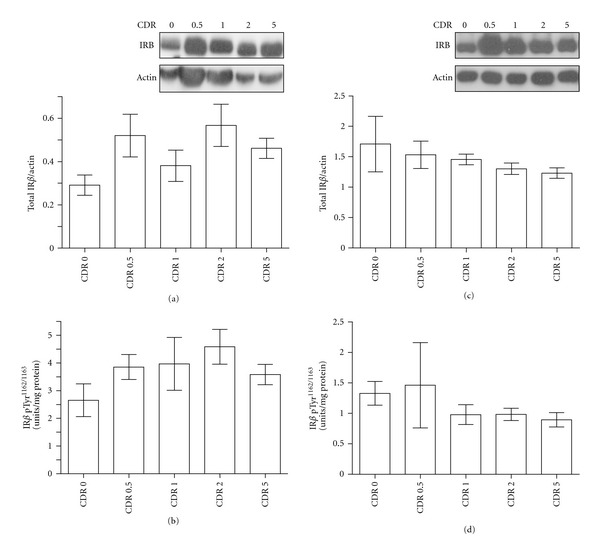
Total IR*β* and [Tyr1162/1163]-phosphorylated IR*β* contents in peripheral temporal muscle and in brain hippocampal formation specimens as a function of CDR. Total insulin IR*β* contents in temporal muscle (a) and in the hippocampal formation (c) were assessed by western blot analysis using a commercial antibody that is selective for IR*β* and is not cross-reactive with IGF-1R*β*. Contents of [Tyr1162/1163]-phosphorylated IR*β* in temporal muscle (b) and in the hippocampal formation (d) were assessed by a commercial ELISA that is specific for [Tyr1162/1163]-phosphorylated IR*β*, and is not cross-reactive with IGF-IR*β*. In ((a) and (c)), total IR*β* contents are expressed relative to *β*-actin levels assessed on the same Western blot using a specific *β*-actin antibody (Sigma, MO). Inset: representative western blot analysis of total IR*β* and *β*-actin contents in muscle ((a), inset) and hippocampal formation ((c), inset) from CDR 0, 0.5, 1, 2, and 5 cases. In ((b) and (d)), [Tyr1162/1163]-phosphorylated IR*β* is expressed relative to total protein contents. In ((a)–(d)), values represent group mean ± SEM values. ANOVA; *P* = .976 and  .478, respectively, for IR*β* and [Tyr1162/1163]-phosphorylated IR*β* in temporal muscle; *P* = .220 and  .425, respectively, for IR*β* and [Tyr1162/1163]-phosphorylated IR*β* in the hippocampal formation.

Consistent with the selection of nondiabetic cases for this study, we found no difference in the contents of total IR*β* (Figure 2(a); ANOVA, *P* = .976) and [Tyr1162/1163]-phosphorylated IR*β* (Figure 2(b); ANOVA, *P* = .478) in peripheral temporal muscle across the CDR groups. Interestingly, comparable findings were also observed in the brains of nondiabetic AD cases. We found no significant difference in the contents of total IR*β* (Figure 2(c); ANOVA, *P* = .220) and [Tyr1162/1163]-phosphorylated IR*β* (Figure 2(d); ANOVA, *P* = .425) in the hippocampal formation across the CDR groups among the nondiabetic cases assessed in this study.

### 3.4. Lack of Correlation between Total IR*β* and [Tyr1162/1163]-Phosphorylated IR*β* Contents in the Hippocampal Formation and AD Neuropathology

**Figure 3:**
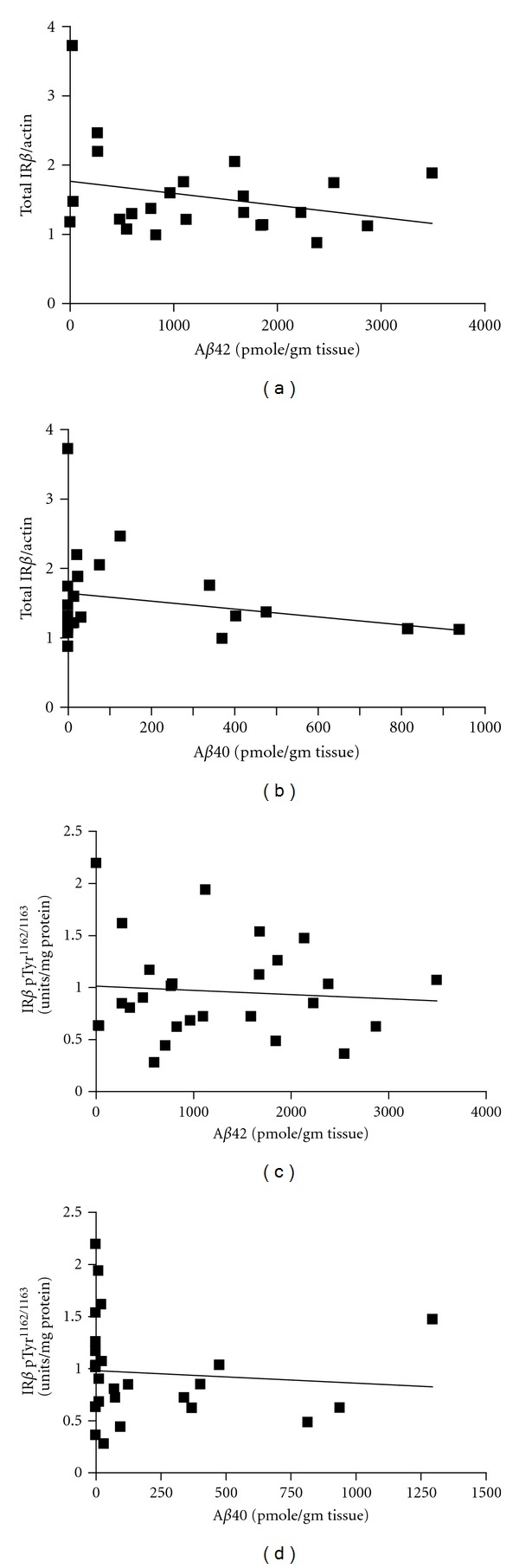
Total IR*β* and [Tyr1162/1163]-phosphorylated IR*β* contents in the hippocampal formation are not correlated with the contents of A*β* peptides. In ((a) and (b)), correlation analysis of total IR*β* content with contents of A*β*_1–42_ (a) and A*β*_1–40_ (b) peptides in the hippocampal formation. In ((c) and (d)), correlation analysis of [Tyr1162/1163]-phosphorylated IR*β* contents with A*β*_1–42_ (c) and A*β*_1–40_ (d) peptides in the hippocampal formation. In ((a)–(d)), solid line represents the best-fit correlation between IR*β* or [Tyr1162/1163]-phosphorylated IR*β* with *β*_1–42_ or A*β*_1–40_ peptides. Pearson correlation analysis; *P* = .205 and  .271 for IR*β* contents with A*β*_1–42_ and A*β*_1–40_, respectively; *P* = .684 and  .681 for [Tyr1162/1163]-phosphorylated IR*β* contents with A*β*_1–42_ and A*β*_1–40_, respectively.

We continued to explore potential interrelationships between total IR and [Tyr1162/1163]-phosphorylated IR*β* contents in the brain and AD neuropathology among the nondiabetic cases. We found no correlation between total IR*β* content and the contents of A*β*_1–42_ (Figure 3(a); *P* = .205) or A*β*_1–40_ (Figure 3(b); *P* = .271) peptides in the hippocampal formation. More importantly, we found that the content of [Tyr1162/1163]-phosphorylated IR*β* in the hippocampal formation is not correlated with the contents of A*β*_1–42_ (Figure 3(c); *P* = .684) or A*β*_1–40_ (Figure 3(d); *P* = .681) peptides.

**Figure 4:**
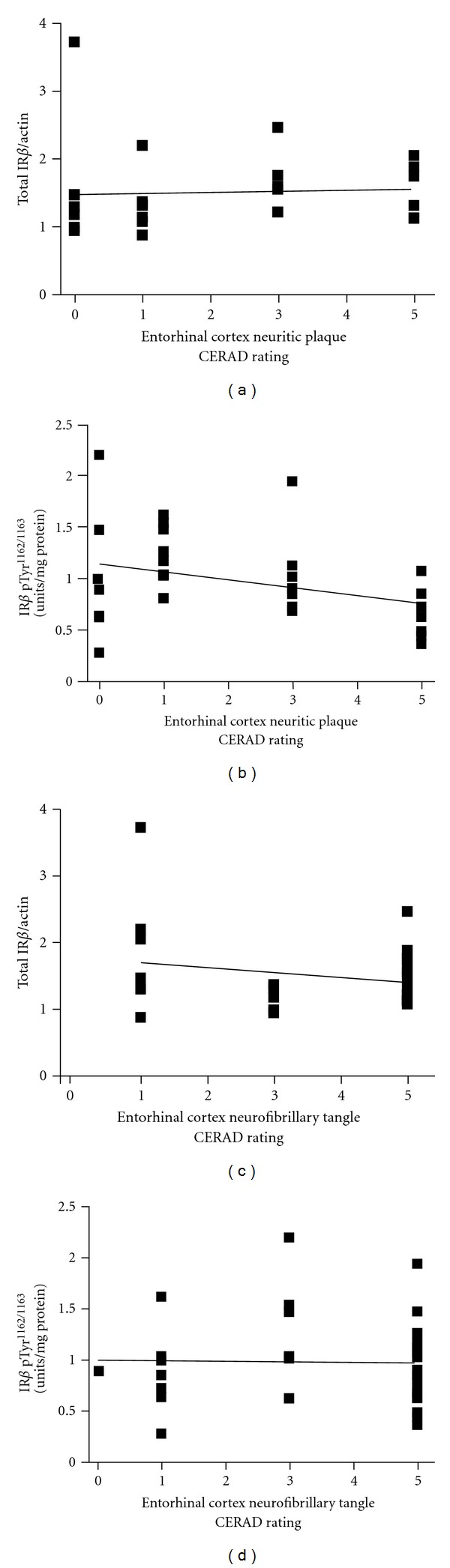
Total IR*β* and [Tyr1162/1163]-phosphorylated IR*β* contents in the brain are not correlated with the severity of AD-type neuropathology. AD-type neuritic plaque (NP) and neurofibrillary tangle (NFT) neuropathology were assessed using CERAD rating scales. In ((a) and (c)), correlation analysis of total IR*β* content with NP (a) and NFT (c) neuropathology in the brain. In ((b) and (d)), correlation analysis of [Tyr1162/1163]-phosphorylated IR*β* contents with NP (b) and NFT (d) neuropathology. In ((a)–(d)), solid line represents the best-fit correlation between IR*β* or [Tyr1162/1163]-phosphorylated IR*β* with NP or NFT neuropathology. Pearson correlation analysis; *P* = .749 and  .516 for IR*β* contents with NP and NFT neuropathology, respectively; *P* = .283 and  .912 for [Tyr1162/1163]-phosphorylated IR*β* contents with NP and NFT neuropathology, respectively.

Consistent with our observation that AD-dementia in nondiabetic cases is not associated with significant changes in the contents of total IR*β* or [Tyr1162/1163]-phosphorylated IR*β* in the brain (Figures 2(c)-2(d)), we found that total or [Tyr1162/1163]-phosphorylated IR*β* contents are not correlated with AD-type amyloid neuritic plaque (NP) or neurofibrillary tangle (NFT) neuropathology in the brain (Figures 4(a)–4(d)). In particular, based on histological assessments of neuritic plaques and neurofibrillary tangles using the 4-point CERAD rating, we found no correlation between the content of total IR*β* in the hippocampal formation and either NPs (Figure 4(a); *P* = .749) or NFTs (Figure 4(c); *P* = .516). Similarly, we found no correlation between the contents of [Tyr1162/1163]-phosphorylated IR*β* in the hippocampal formation and either NPs (Figure 4(b); *P* = .283) or NFTs (Figure 4(d); *P* = .912).

### 3.5. Assessment of IR-Associated Molecular Signaling in the AD Brain

**Figure 5:**
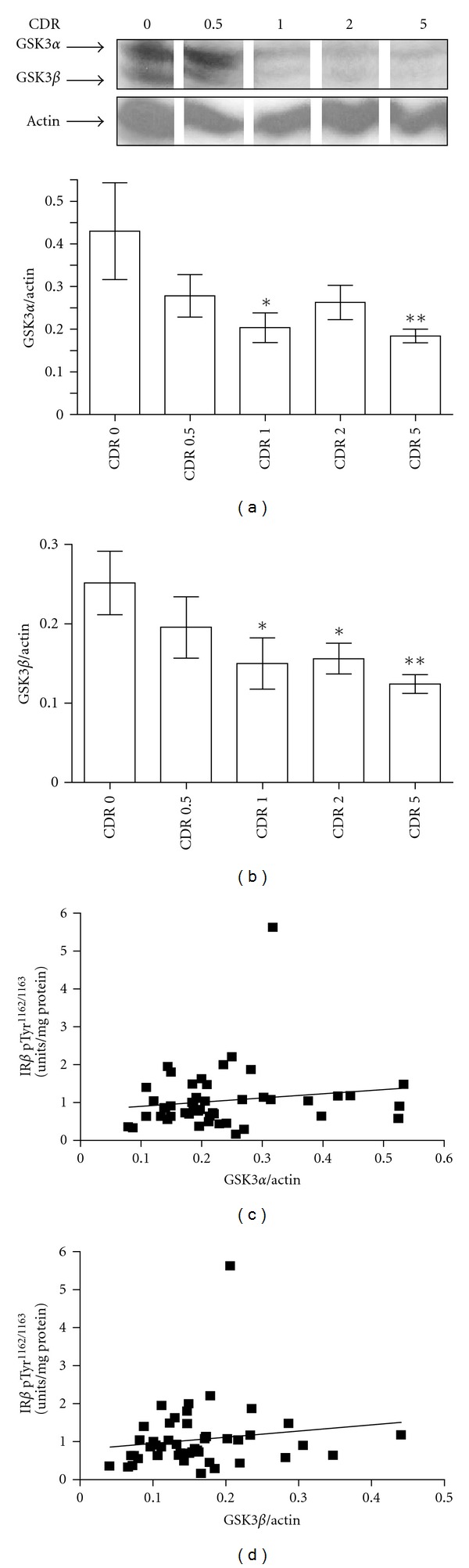
Reduced contents of total GSK3*α* and GSK3*β* in the hippocampal formation in AD brain specimens are not correlated with [Tyr1162/1163]-phosphorylated IR*β*. Total GSK3*α* and GSK3*β* contents in the hippocampal formation were assessed by Western blot analysis. In ((a) and (b)), bar graphs represent mean ± SEM total GSK3*α* (a) and total GSK3*β* (b) contents (nonphosphorylated and phosphorylated GSK3 *α*/*β*) in the hippocampal formation relative to *β*-actin as a function of Clinical Dementia Rating. ANOVA; *P* = .0111 and  .0112, respectively, for GSK3*α* and GSK3*β* contents among CDR groups. One-tailed *t*-test in comparison to CDR 0: *P < .05; **P < .005. Inset: representative Western blot analysis of total GSK3*α* and total GSK3*β* from CDR 0, 0.5, 1, 2 and 5 hippocampal formation specimens. In ((c) and (d)), correlation analysis of GSK3*α* (c) and GSK3*β* (d) contents with respect to [Tyr1162/1163]-phosphorylated IR*β* contents in the hippocampal formation. Pearson correlation analysis; *P* = .318 and  .308 for GSK3*α* and GSK3*β* contents with contents of [Tyr1162/1163]-phosphorylated IR*β*, respectively.

Numerous studies have documented changes in IR-responsive cellular signaling pathways in the brain. For example, data has shown reduced GSK3 *α* and *β* contents and activities [24, 25] in the AD brain. Consistent with these observations, we observed significantly lower contents of total GSK3*α* (Figure 5(a); *P* < .05) and GSK3*β* (Figure 5(b); *P* < .005) in the hippocampal formation of CDR 1, 2 and 5 cases in comparison to neurological control (CDR 0) cases. Interestingly, we found no correlation between the contents of [Tyr1162/1163]-phosphorylated IR*β* and either total GSK3*α* (Figure 4(c); Pearson Correlational analysis, *P* = .318) or total GSK3*β* (Figure 4(d); Pearson Correlation analysis, *P* = .308) in the hippocampal formation. Thus, our evidence suggests that downregulation of total GSK3 *α*/*β* contents in the brains of the nondiabetic AD cases analyzed in this study might be mediated by mechanisms independent of IR activation.

## 4. Discussion

Recent hypotheses raised the possibility that impaired IR signaling in the brain might be a common underlying cause of sporadic AD [19, 23, 41]. Although cellular IR activation is the first, and a necessary, step in cellular IR-signaling processes, there is no consensus on the regulation of IR content and IR activation in the brains of sporadic AD cases [19, 21, 23]. With the exception of a recent publication by Liu et al., [18], it is not known whether any of the AD and control cases used in previously reported studies are characterized by T2D. It is possible that the outcomes in these reports might be complicated by inclusion of T2D cases. The recent publication by Liu et al. [18] reported no significant change in IR*β* levels in the brains of nondiabetic AD cases, but did not report the status of IR activation.

This study was designed to investigate the contents of IR*β* and [Tyr1162/1163]-phosphorylated IR*β* as surrogate indices of, respectively, total IR contents and IR activation in the brains of nondiabetic AD cases as a function of AD dementia and AD-type neuropathology. Among the nondiabetic cases examined in this study, we found that total IR*β* contents in postmortem hippocampal specimens from cases characterized by mild cognitive impairment (CDR 0.5), mild AD dementia (CDR 1), moderate AD dementia (CDR 2) and severe AD dementia (CDR 5) were comparable to levels that were found in cognitive normal (CDR 0) control cases. Our findings are consistent with observations by Moloney et al. [23] and Liu et al. [18], who reported comparable levels of total IR*α* and IR*β* proteins in postmortem temporal cortex specimens from severe AD and control cases. In addition to total IR*β* protein contents, evidence from our nondiabetic cohort also revealed similar levels of [Tyr1162/1163]-phosphorylated IR*β* in hippocampal specimens from CDR 0.5, 1, 2, and 5 cases compared to control CDR 0 cases. Moreover, we found that the severity of amyloid and tau AD-neuropathology among nondiabetic AD cases was not correlated with the contents of either total IR*β* or [Tyr1162/1163]-phosphorylated IR*β* in the hippocampal formation. Collectively, our observations tentatively suggest that nondiabetic sporadic AD is characterized by normal IR content and IR activation in the brain. Interestingly, Moloney et al. [23] observed aberrant subcellular distributions of IR*α* and IR*β* proteins among surviving neurons in brain specimens from severe AD cases, without the consideration of the diabetic/nondiabetic status of these cases. Future studies will be necessary to examine whether nondiabetic CDR 0.5, 1, 2, and 5 cases might also be characterized by similar aberrant subcellular distribution of IR*α*/*β* and [Tyr1162/1163]-phosphorylated IR*β* in the brain.

Activation of the IR leads to the modulation of a large number of cellular signaling processes [42–44]. However, many of these cellular signaling molecules such as Akt and GSK3 *α*/*β* are also regulated by other signaling processes [45–49]. For example, activation of IR or insulin-like growth factor 1 receptor (IGF-1R) both lead to receptor-mediated tyrosine phosphorylation of adaptor proteins such as insulin receptor substrate proteins that, in turn, modulate the activation of Akt [45], GSK3 [50, 51], extracellular signal-regulated kinase (ERK) [52], and other signaling pathways. Accumulating epidemiological and experimental evidence suggests that impairments in select IR-associated cellular signaling pathways in the AD brain might mechanistically promote the AD phenotype [2, 3, 6, 7, 14–17, 25]. Among cellular processes that are typically associated with IR-signaling, impaired GSK3 *α*/*β* function in the AD brain is considered pivotal for the development of AD [24–26, 53].

Consistent with previous reports [24, 25], we observed significantly lower levels of total GSK3 *α*/*β* in brain specimens from nondiabetic sporadic AD cases examined in this study. Our observation is consistent with evidence from Griffin et al. [25], which demonstrated increased Akt activation coinciding with elevated levels of inactive Ser9-phosphorylated GSK-3*β* in the temporal cortex of AD cases. IR (as well as the IGF-1R) signaling pathways are known to regulate Akt, GSK3 *α*/*β* and other signal transduction mediators, primarily by modulating the phosphorylation status and thereby the activities of these signal transduction components [50–52, 54]. Based on this consideration and on our observation suggesting normal IR contents and IR activation in brain specimens from our study cohort, downregulation of total GSK3 *α*/*β* contents in the brains of nondiabetic sporadic AD cases is likely mediated by mechanisms independent of IR activation. Additional studies will be necessary to clarify whether there might be changes in the regulation of other IR-associated cellular signaling mechanisms in the brains of nondiabetic cases, and the mechanisms by which cellular contents and activities of Akt, GSK3 *α*/*β*, and other IR mediators might be modulated in the AD brain. Nonetheless, consistent with a recent report by Moloney et al. [23], our observation suggests that, in spite of our evidence suggesting normal IR contents and IR activation, impaired IR signaling mechanisms in the brains of nondiabetic sporadic AD cases might contribute to the onset and/or progression of AD dementia.

Numerous epidemiological studies have linked T2D with an increased risk for AD [2, 3, 6, 7]. We [14] and others [15] demonstrated that diet-induced T2D in the Tg2576 AD mouse model leads to the promotion of AD-type amyloid neuropathology and cognitive deterioration, which are both associated with impaired IR activity and IR signaling the brain. While our present studies suggest the existence of impaired IR signaling in the brains of nondiabetic sporadic AD cases, a recent study by Liu et al. [18] suggests that AD and T2D may induce impaired IR signaling in the brain via different mechanisms than those implicated in our studies, and that the presence of T2D may exacerbate IR signaling impairments in the AD brain.

There is an increasing effort to develop novel AD therapeutics based on the promotion of IR-signaling processes by either directly inducing IR activation (e.g., nasal insulin inhalation [55–57]) or by applying insulin-sensitization measures (e.g., PPAR*γ* activators [41, 58, 59]) that stimulate downstream IR-signaling. Prior studies have not yet explored the potential impact of comorbid diabetic versus nondiabetic conditions on the regulation of IR activation in the AD brain. Results from our study demonstrating reduced contents of total GSK3 *α*/*β* in the brains of nondiabetic sporadic AD cases suggest that, even in the absence of comorbid diabetic conditions, impaired downstream IR signaling processes in the AD brain may contribute to the onset and/or progression of AD phenotypes. This would support the application of insulin-sensitization therapeutic strategies in nondiabetic, sporadic AD. While accumulating experimental evidence suggests that diabetic conditions could lead to reduced IR activity in the brain [14, 15], our present study found no detectable changes in IR activity in the brains of nondiabetic sporadic AD cases. Based on this, we suggest that, in comparison to nondiabetic sporadic AD cases, sporadic AD cases with concomitant diabetic conditions may respond better to therapeutic strategies such as intranasal insulin administration that are designed to directly target IR in the brain.

## Figures and Tables

**Table 1:** Characteristics of study subjects^1^.

	No. of	Mean ± SEM	Mean ± SEM		Median CERAD	Median CERAD
CDR Score	subjects	Postmortem interval, h	Age, y	Female, %	Plague rating	Tangle rating
0	10	5.44 ± 0.91	80.40 ± 6.02	70%	0	0
0.5	9	4.30 ± 0.40	87.33 ± 2.59	67%	3	0
1	11	4.63 ± 0.69	85.00 ± 3.33	55%	3	0
2	13	4.89 ± 0.87	87.08 ± 1.92	85%	5	3
5	19	4.82 ± 0.72	83.06 ± 2.37	74%	5	3

^1^Only nondiabetic cases are selected for this study; cases with a premorbid history of diabetes are excluded. Subjects are grouped by Clinical Dementia rating (CDR). Neuropathology is assessed using Consortium to Establish A Registry for Alzheimer's Disease (CERAD) ratings. Age and postmortem interval (PMI) are in years.

## Abbreviations

A*β*: Beta-amyloid
AD: Alzheimer's disease
APP: Amyloid precursor protein
CDR: Clinical Dementia Rating
CERAD: Consortium to Establish a Registry for Alzheimer's disease
ELISA: Enzyme-linked immunosorbent assay
GSK3: Glycogen synthase kinase 3
IDE: Insulin-degrading enzyme
IGF-1R: Insulin-like growth factor-1 receptor
IGF-1R*β*: IGF-1R beta subunit
IR: Insulin receptor
IR*β*: IR beta subunit
NFT: Neurofibrillary tangles
T2D: Type 2 diabetes
NP: Neuritic plaque.
